# Gender specific association of parathyroid hormone and vitamin D with metabolic syndrome in population with preserved renal function

**DOI:** 10.1038/s41598-017-17397-9

**Published:** 2018-01-18

**Authors:** Min-Hee Kim, Jeongmin Lee, Jeonghoon Ha, Kwanhoon Jo, Dong-Jun Lim, Jung-Min Lee, Sang-Ah Chang, Moo-Il Kang, Bong Yun Cha

**Affiliations:** Department of Internal Medicine, Division of Endocrinology and Metabolism, College of Medicine, The Catholic University of Korea, Seoul, Republic of Korea

## Abstract

The association of parathyroid hormone (PTH) and vitamin D with Metabolic syndrome (MetS) was evaluated using representative data from the Korean population. Data from 7004 subjects aged 50 or older with preserved renal function (excluding chronic kidney disease stage 3b to 5) who were included in the Korean National Health and Nutrition Examination Survey between 2008 and 2010 were analysed. Higher PTH levels (pg/ml) were observed in subjects with MetS than in those without MetS among both genders (60.1 (58.6–61.6) vs. 62.4 (60.7–64.2) in males *p* = *0.018*, 60.7 (59.4–62.1) vs. 63.9 (62.4–65.6) in females, *p* < *0.001*). For females, PTH levels were significantly higher in subjects with MetS than in those without MetS after adjustment for possible covariates. Lower 25(OH)D levels were significantly associated with MetS only in male subjects (*p* = *0.004*). As the number of MetS components increased, a significant rise in PTH levels (*p* for trend 0.005 in males and 0.024 in females) and a decrease in 25(OH)D levels (*p* for trend < 0.001 in males and 0.053 in females) were observed. In conclusion, among subjects with preserved renal function, PTH levels were possibly associated with MetS in females, whereas vitamin D levels exhibited a possible link to MetS in males.

## Introduction

Parathyroid hormone (PTH) and vitamin D are the primary regulators of calcium homeostasis. In addition to their conventional role, both have been associated with insulin resistance in subjects with and without primary hyperparathyroidism (PHP)^1^, and an inverse relationship between PTH and insulin sensitivity was observed^2^. Vitamin D has also been suggested to be related to beta cell function^3^, and insulin resistance is related to vitamin D status^4,5^.

Metabolic syndrome (MetS), a cluster of symptoms that include hypertension, high blood glucose and triglyceride levels, low high-density lipoprotein (HDL) cholesterol, and increased waist circumference, is suspected to be related to PTH status or vitamin D levels, as insulin resistance is an underlying mechanism. Several studies have evaluated the relationship between PTH/vitamin D and MetS in large population-based datasets^4,6–15^, but the results were inconsistent. This inconsistency may be due to the heterogeneity of the included study populations and confounding factors in the analysis. Specifically, with the exception of two studies^6,10^, the aforementioned large-scale studies did not include exclusion criteria for subjects with reduced glomerular filtration rates (GFR). Recent results from the Korean National Health and Nutrition Examination Survey (KNHANES) had no exclusion criteria for subjects with renal impairment, and the status of renal function based on the GFR was not considered in the evaluation of the relationship between PTH/vitamin D and MetS^15^.

However, the status of both PTH and vitamin D is closely related to impaired renal function. For example, a steep increase in serum PTH levels during moderate chronic kidney disease (CKD) (GFR <60 ml/min/1.73 m^2^) is well known^16^. Vitamin D deficiency, which is a stimulator of PTH secretion, has been frequently observed in subjects with CKD^7^. As an insufficient vitamin D status in CKD subjects is associated with unfavourable outcomes, vitamin D supplementation along with calcium in cases of secondary hyperparathyroidism is recommended by clinical guidelines^17^. The incidence of MetS was also observed to increase with a decline in the GFR in a study examining a nationally representative population^18^. Besides CKD 4 and 5, CKD stage 3b (GFR <45 ml/min/1.73 m^2^) patients exhibited an increased risk of MetS compared to stage 3a (45 ≤ GFR <60 ml/min/1.73 m^2^) patients^19^. Therefore, exclusion of subjects with impaired renal function is necessary for the evaluation of the association of PTH/vitamin D with MetS. In addition, because gender could affect body composition changes during ageing^20^ and calcium homeostasis^21^, and both of these criteria can possibly influence PTH and vitamin D, the associations of PTH and vitamin D with MetS were evaluated separately for each gender.

The aim of this study is to elucidate the associations of PTH and vitamin D with MetS in subjects with normal kidney function using representative datasets of the Korean population.

## Methods

### Study population

The study examined data from subjects aged 50 years or older from the fourth and fifth KNHANES (n = 10,238) collected from 2008 to 2010. The survey was cross-sectional and representative of non-institutionalized Korean people. The KNHANES, which was designed based on stratified multistage probability sampling, is conducted by the Korean Centre for Disease Control and Prevention. The detailed design of sampling and variables are described elsewhere^22^. The collected data consisted of information about health and nutritional status as well as socioeconomic status. Subjects with fasting times less than 8 hours before laboratory tests, treatment for osteoporosis, or missing covariates were excluded. In addition, subjects with stage 3b to 5 CKD (GFR ≤45 ml/min/1.73 m^2^, moderate to severe CKD) were excluded from the study. Finally, data from 7004 subjects were analysed. All participants provided written informed consent for the survey, and the study protocol was approved by Institutional Review Board of The Catholic University of Korea (No. PC16EISI0037). The study was conducted based on the Declaration of Helsinki for biomedical research.

### Demographic characteristics and Survey on status of health and nutrition

Demographic data and medical histories were obtained from self-reported questionnaires and personal interviews by trained personnel. A 24-hour recall method and Can-Pro 2.0 nutrient intake assessment software, developed by the Korean Nutrition Society, were used for assessment of dietary intake and daily energy intake, respectively. Regular exercise was defined as exercise for at least 20 minutes 3 times per week with moderate to severe intensity.

Drinking status was classified as non-to-moderate drinking and heavy drinking. Heavy drinking was defined as intake of more than 30 g/day of alcohol. Smoking status was divided into current smokers and non-smokers (including ex-smokers). Low educational status was defined as those with elementary to middle school as their highest academic achievement. The lowest quartile of income was defined as low income.

### Anthropometric and laboratory measurements

Height, body weight, and waist circumference were measured by a standardized protocol^23^. Briefly, waist circumference was defined as the distance from the midline of the lower rib margin to the iliac crest. The weight and height of subjects were measured with light clothing after removing their shoes. The weight (kg) per square of height (m^2^) was calculated as the BMI. Blood pressure was measured three times using a mercury sphygmomanometer (Baumanometer; W.A. Baum Co. Inc., Copiague, NY, USA) with patients in a seated position after 5 minutes of rest.

For laboratory measurements, all subjects fasted for at least 8 hours before sampling. The samples were processed immediately and then refrigerated for transportation to the central testing institute (NeoDin Medical Institute, Seoul, Korea). Detailed information about measurement methods for laboratory parameters including glucose, cholesterol, PTH and serum 25-hydroxyvitamin D (25(OH)D), which reflects the status of vitamin D, were described in previous studies^5,15^. The Friedewald formula was used for the calculation of low-density lipoprotein (LDL) cholesterol^24^. The modification of diet in renal disease (MDRD) equation was used to calculate the estimated GFR (eGFR)^25^.

### Definition of metabolic syndrome and its components

The criteria for the presence of MetS were determined based on the criteria suggested by the American Heart Association (AHA)/National Heart, Lung, and Blood Institute (NHLBI) and the World Health Organization (WHO) Asia–Pacific region criteria for abdominal obesity. Specifically, MetS was defined when subjects had at least 3 of the 5 following components:

1. Waist circumference ≥90 cm for men and ≥80 cm for women
2. Triglycerides ≥150 mg/dl
3. HDL cholesterol <40 mg/dl for men and <50 mg/dl for women
4. Blood pressure ≥130/85 mmHg or current use of antihypertensive medication
5. Fasting glucose ≥100 mg/dl or current use of antidiabetic medication/insulin

When subjects were reported to take medication for dyslipidemia, it was regarded as the presence of both high triglyceride and low HDL cholesterol levels.

### Statistical analysis

To provide nationally representative prevalence estimates, statistical procedures were performed to reflect the complex sampling design and sampling weights of the KNHANES. The SAS PROC SURVEY module was used to consider strata, clusters, and weights. Based on the characteristics of the data, the results were expressed as the means ± standard error (SE), geometric means (95% confidence interval [CI]) or percentages ( ± SE), as appropriate. Characteristics of each gender were compared with the chi-squared test for dichotomous variables and independent t-tests for continuous variables. Logarithmic transformations of variables with skewed distributions were performed before statistical analysis. To compare the mean of PTH and vitamin D levels between groups according to the absence or presence of MetS and each of its components, analysis of covariance (ANCOVA) was performed to adjust several covariates. Multivariate logistic regression analysis for the presence of metabolic syndrome was performed. To acquire clinical significance from the analysis, the population was categorized into two groups; population with the 1^st^ to 3^rd^ vs. 4^th^ quartiles of PTH levels (cut-off values for men: 76.8 pg/ml, for female: 77.6 pg/ml) and those with less than 20 ng/ml vs. 20 ng/ml or above of vitamin D levels. All statistical analyses were performed using SAS version 9.3 software (SAS Institute Inc., Cary, NC, USA).

## Results

### Baseline characteristics of the population

The mean ages of male and female participants were 60.8 ± 0.2 and 61.7 ± 0.2 years, respectively (Table 1). In contrast to incidence of diabetes, MetS was more frequently observed in females (52.3%) than in males (43.1%). Although the intake of calcium was significantly higher in females than that in males, significantly increased levels of PTH and decreased levels of 25(OH)D were observed in female subjects compared to male subjects. Other parameters including MetS components and socioeconomic status are shown in Table 1.

**Table 1:** Baseline characteristics total population

	Male (n = 3190)	Female (n = 3814)	P-value
Age (years)	60.8 ± 0.2	61.7 ± 0.2	< 0.0001
Smoking (%)	35.5(1.1)	4.8(0.5)	< 0.0001
Drinking (%)	17.8(0.9)	0.8(0.2)	< 0.0001
Regular exercise (%)	28.3(1.1)	23.8(1)	0.0004
Low Education status (%)	46.9(1.3)	23.3(1.1)	< 0.0001
Low income (%)	23.7(1)	31.3(1.1)	< 0.0001
Calcium intake (mg)	431.5 + 8.4	559.0 + 9.2	< 0.0001
DM (%)	18.1(0.8)	14.4(0.7)	0.0008
HTN (%)	51.6(1.2)	49.1(1)	0.103
Metabolic syndrome (%)	43.1(1.1)	52.3(1.1)	< 0.0001
BMI (kg m^−2^)	23.9 ± 0.1	24.3 ± 0.1	< 0.0001
Waist circumference (cm)	85.4 ± 0.2	82.3 ± 0.2	< 0.0001
Systolic blood pressure (mmHg)	127.2 ± 0.4	126.8 ± 0.4	0.5328
Diastolic blood pressure (mmHg)	81 ± 0.3	78.2 ± 0.2	< 0.0001
Fasting glucose (mmol l^−1^)	104.8 ± 0.5	101.1 ± 0.5	< 0.0001
Total cholesterol (mmol l^−1^)	186.7 ± 0.8	201.4 ± 0.7	< 0.0001
Triglyceride (mmol l^−1^)	133.9(130.3–137.6)	118.5(115.9–121.2)	< 0.0001
HDL cholesterol (mmol l^−1^)	48.4 ± 0.3	52.5 ± 0.3	< 0.0001
LDL cholesterol(mmol l^−1^)	112 ± 0.7	126.1 ± 0.6	< 0.0001
Glomerular filtration rate (	91.37 + 0.4	88.9 ± 0.4	< 0.0001
PTH (pg/mL)	61.1(59.8–62.4)	62.4(61.2–63.6)	0.0457
Serum 25(OH)D (ng/mL)	21.6 + 0.3	18.5 + 0.2	< 0.0001

BMI, body mass index; LDL, low-density lipoprotein; HDL, high-density lipoprotein; HOMA-IR, homeostasis model assessment of insulin resistance; PTH, parathyroid hormone; Normal range of PTH is 8–76 pg/mL. Data are expressed as the means ± standard error (SE), percentage (SE) or geometric means (95% confidence interval).

Low educational status: subject with elementary to middle school.

Low income: the lowest quartile.

### Comparison of PTH and vitamin D levels of subjects with and without MetS (and its components)

Higher PTH levels were observed in populations with MetS than in those without MetS among both genders (model 1 in Table 2). For females, PTH levels were significantly higher in subjects with MetS than in those without MetS after adjustment for possible covariates (model 2–4 in Table 2). In contrast to PTH levels, differences in 25(OH)D levels were observed only in male subject groups (model 1–3 in Table 3)

**Table 2:** Comparison of parathyroid hormone levels between groups according to presence or absence of metabolic syndrome or its components.

	MODEL 1		MODEL 2		MODEL 3		MODEL 4	
	Male	Female	Male	Female	Male	Female	Male	Female
Metabolic syndrome								
Absent	60.1(58.6–61.6)	60.7(59.4–62.1)	60.3(58.8–61.9)	61.8(60.4–63.2)	60.3(58.7–61.9)	61.5(60.1–62.9)	60.4(58.8–62)	61.3(59.9–62.7)
Present	62.4(60.7–64.2)	63.9(62.4–65.6)	62.7(60.9–64.5)	63.6(62–65.2)	62(60.2–63.8)	63.5(61.8–65.2)	61.6(59.9––63.4)	63.2(61.6–64.7)
P value	0.0188	0.0003	0.0188	0.05	0.1066	0.0394	0.2289	0.0493
High waist circumference								
Absent	60.9(59.6–62.2)	59.9(58.4–61.4)	61.1(59.8–62.4)	60.7(59.1–62.2)	61(59.6–62.4)	60.4(58.8–62)	60.8(59.5–62.2)	60.2(58.7–61.8)
Present	61.6(59.4–63.9)	64.1(62.6–65.6)	61.8(59.6–64.1)	64.1(62.7–65.7)	61.1(59–63.3)	64(62.4–65.7)	61.1(59–63.3)	63.7(62.2–65.2)
P value	0.5032	< 0.0001	0.5042	0.0005	0.9189	0.0006	0.7687	0.0008
High blood pressure								
Absent	58.7(57–60.5)	58.4(56.9–59.8)	59(57.3–60.8)	59.5(58.1–61.1)	58.7(56.8–60.6)	59(57.5–60.6)	58.9(57–60.8)	59(57.5–60.5)
Present	62.5(61–64)	65.3(63.8–66.8)	62.6(61.2–64.1)	64.9(63.4–66.4)	62.4(60.9–63.9)	64.9(63.3–66.5)	62.1(60.6–63.5)	64.4(63–65.9)
P value	0.0001	<0.0001	0.0002	< 0.0001	0.0005	< 0.0001	0.0026	< 0.0001
High blood gluose								
Absent	60.9(59.2–62.7)	61.9(60.6–63.3)	61.2(59.5–62.9)	62.6(61.3–64)	60.9(59.3–62.7)	62.6(61.2–64.1)	61(59.4–62.7)	62.4(61–63.8)
Present	61.2(59.7–62.8)	63.1(61.4–64.9)	61.4(59.9–63.1)	62.9(61.2–64.7)	61.1(59.5–62.8)	62.5(60.7–64.3)	60.8(59.2–62.4)	62.2(60.5–63.9)
P value	0.7611	0.2066	0.8005	0.7557	0.8553	0.8693	0.8275	0.8544
Hypertriglyceridemia								
Absent	60.6(59.2–62.1)	62.8(61.5–64.2)	60.8(59.4–62.2)	63.5(62.2–64.9)	60.9(59.5–62.5)	63.3(61.9–64.7)	61(59.6–62.5)	63.1(61.8–64.5)
Present	61.7(59.8–63.6)	61.7(59.9–63.5)	62(60.1–64)	61.5(59.8–63.4)	61.1(59.3–63.1)	61.5(59.6–63.4)	60.8(59–62.6)	61(59.2–62.8)
P value	0.3086	0.238	0.2334	0.0532	0.8485	0.0951	0.8349	0.0345
Low HDL cholesterol								
Absent	60.2(58.7–61.8)	61.7(60.1–63.4)	60.5(58.9–62.1)	62.8(61.2–64.5)	60(58.4–61.7)	62.8(61.1–64.6)	60.2(58.6–61.8)	62.8(61.1–64.5)
Present	62.2(60.5–63.9)	62.7(61.4–64.1)	62.5(60.8–64.2)	62.7(61.4–64.1)	62.3(60.6–64.1)	62.4(61–63.9)	61.9(60.2–63.6)	62(60.7–63.4)
P value	0.0406	0.2837	0.042	0.9316	0.0281	0.6669	0.092	0.448

MODEL 1 non–adjusted.

MODEL 2 age, sex.

MODEL 3 age, sex, smoke, exercise, education levels, income status, calcium intake.

MODEL 4 age, sex, smoke, exercise, education levels, income status, calcium intake, vitamin D.

**Table 3:** Comparison of vitamin D levels between groups according to presence or absence of metabolic syndrome or its components.

	MODEL 1		MODEL 2		MODEL 3		MODEL 4	
	Male	Female	Male	Female	Male	Female	Male	Female
Metabolic syndrome								
Absent	22 ± 0.3	18.7 ± 0.2	22.1 ± 0.3	18.6 ± 0.2	22.1 ± 0.3	18.7 ± 0.3	22 ± 0.3	18.6 ± 0.3
Present	21.1 ± 0.3	18.4 ± 0.3	21.2 ± 0.3	18.4 ± 0.3	21.4 ± 0.3	18.4 ± 0.3	21.4 ± 0.3	18.5 ± 0.3
P value	0.0044	0.3512	0.0045	0.4098	0.0312	0.4767	0.0613	0.7903
High waist circumference								
Absent	21.6 ± 0.3	18.5 ± 0.3	21.6 ± 0.3	18.5 ± 0.3	21.7 ± 0.3	18.7 ± 0.3	21.7 ± 0.3	18.5 ± 0.3
Present	21.7 ± 0.4	18.5 ± 0.3	21.8 ± 0.4	18.5 ± 0.3	22 ± 0.4	18.5 ± 0.3	22 ± 0.4	18.6 ± 0.3
P value	0.5612	0.9223	0.5602	0.97	0.2814	0.5441	0.271	0.8591
High blood pressure								
Absent	22.2 ± 0.3	18.9 ± 0.3	22.3 ± 0.4	19 ± 0.3	22.4 ± 0.3	19 ± 0.3	22.2 ± 0.3	18.8 ± 0.3
Present	21.3 ± 0.3	18.3 ± 0.2	21.3 ± 0.3	18.2 ± 0.3	21.4 ± 0.3	18.3 ± 0.3	21.5 ± 0.3	18.4 ± 0.2
P value	0.0024	0.009	0.0012	0.0153	0.0039	0.0148	0.0291	0.267
High blood gluose								
Absent	21.8 ± 0.3	18.6 ± 0.2	21.9 ± 0.3	18.6 ± 0.2	22.1 ± 0.3	18.6 ± 0.2	22.1 ± 0.3	18.6 ± 0.2
Present	21.4 ± 0.3	18.4 ± 0.3	21.4 ± 0.3	18.4 ± 0.3	21.4 ± 0.3	18.5 ± 0.3	21.4 ± 0.3	18.5 ± 0.3
P value	0.1961	0.6449	0.1784	0.6955	0.0608	0.8803	0.06	0.8524
Hypertriglyceridemia								
Absent	22 ± 0.3	18.7 ± 0.2	22 ± 0.3	18.7 ± 0.2	22.1 ± 0.3	18.7 ± 0.2	22.1 ± 0.3	18.8 ± 0.2
Present	21.1 ± 0.3	18.2 ± 0.3	21.2 ± 0.3	18.2 ± 0.3	21.3 ± 0.3	18.2 ± 0.3	21.3 ± 0.3	18.2 ± 0.3
P value	0.005	0.0879	0.0077	0.0999	0.0164	0.0572	0.0174	0.014
Low HDL cholesterol								
Absent	22.1 ± 0.3	18.8 ± 0.2	22.1 ± 0.3	18.8 ± 0.2	22.2 ± 0.3	18.7 ± 0.3	22.1 ± 0.3	18.8 ± 0.3
Present	21 ± 0.3	18.4 ± 0.3	21.1 ± 0.3	18.4 ± 0.3	21.2 ± 0.3	18.4 ± 0.3	21.3 ± 0.3	18.5 ± 0.3
P value	0.0016	0.1444	0.0015	0.1603	0.0062	0.339	0.0132	0.2629

MODEL 1 non–adjusted.

MODEL 2 age, sex.

MODEL 3 age, sex, smoke, exercise, education levels, income status, calcium intake.

MODEL 4 age, sex, smoke, exercise, education levels, income status, calcium intake, PTH.

For both genders, subjects with hypertension showed higher PTH levels (model 1–4 in Table 2) and lower 25(OH)D levels (model 1–3 for females, model 1–4 for males in Table 3) than those without hypertension. Higher PTH levels were found in female subjects with the waist circumference component of MetS (model 1–4 in Table 2). For male subjects, those with the HDL cholesterol component (low HDL cholesterol levels) of MetS showed higher PTH and lower 25(OH)D levels than those with normal HDL levels. Male subjects with the triglyceridxe component of MetS presented lower 25(OH)D levels than those without this component.

In multivariate logistic regression analysis (Table 4), gender specific association patterns of PTH and vitamin D with metabolic syndrome were observed. After adjustment of possible confounding factors, the highest quartiles of PTH was significantly associated with increased risk of MetS in females, while vitamin D deficiency (less than 20 ng/ml) was related to increased risk in males.

**Table 4:** Multiple logistic regression analysis (Presence of Metabolic syndrome – PTH or Vitamin D).

	Unadjusted		Adjusted						
	MODEL 1		MODEL 2			MODEL 3		MODEL 4	
	OR (95% CI)		OR (95% CI)			OR (95% CI)		OR (95% CI)	
	Male	Female	Male		Female	Male	Female	Male	Female
PTH*									
1–3^rd^ quartiles	reference	reference		reference	reference	reference	reference	reference	reference
4^th^ quartile	1.24 (1.022–1.505)	1.382 (1.15–1.661)		1.241 (1.022–1.507)	1.213(1.005,1.463)	1.148(0.929,1.417)	1.238(1.012,1.514)	1.105(0.894,1.365)	1.227(1.002,1.504)
P value	0.0295	0.0006		0.0295	0.044	0.201	0.0382	0.3549	0.0482
**Vitamin D**									
Less than 20 ng/ml	reference	reference	reference		reference	reference	reference	reference	reference
20 ng/ml or more	0.795(0.675,0.937)	0.97(0.832,1.132)	0.795(0.675,0.937)		0.963(0.825,1.124)	0.812(0.679,0.97)	0.947(0.798,1.125)	0.824(0.688,0.988)	0.978(0.823,1.163)
P value	0.0062	0.6993	0.0063		0.632	0.0219	0.5378	0.0362	0.8038

Statistics were carried out using Logistic regression. adjusted for age, sex, self-pay of cancer screening, weight variation, waist-height ratio, physical activity, drink, smoking.

MODEL 1 non-adjusted.

MODEL 2 age, sex.

MODEL 3 age, sex, smoke, exercise, education levels, income status, calcium intake.

MODEL 4 age, sex, smoke, exercise, education levels, income status, calcium intake, PTH (or vitamin D).

*The cut-off values for the 4^th^ quartiles of PTH was 76.8 pg/ml in male and 77.6 pg/ml in female.

### Number of MetS components and PTH levels

**Figure 1:**
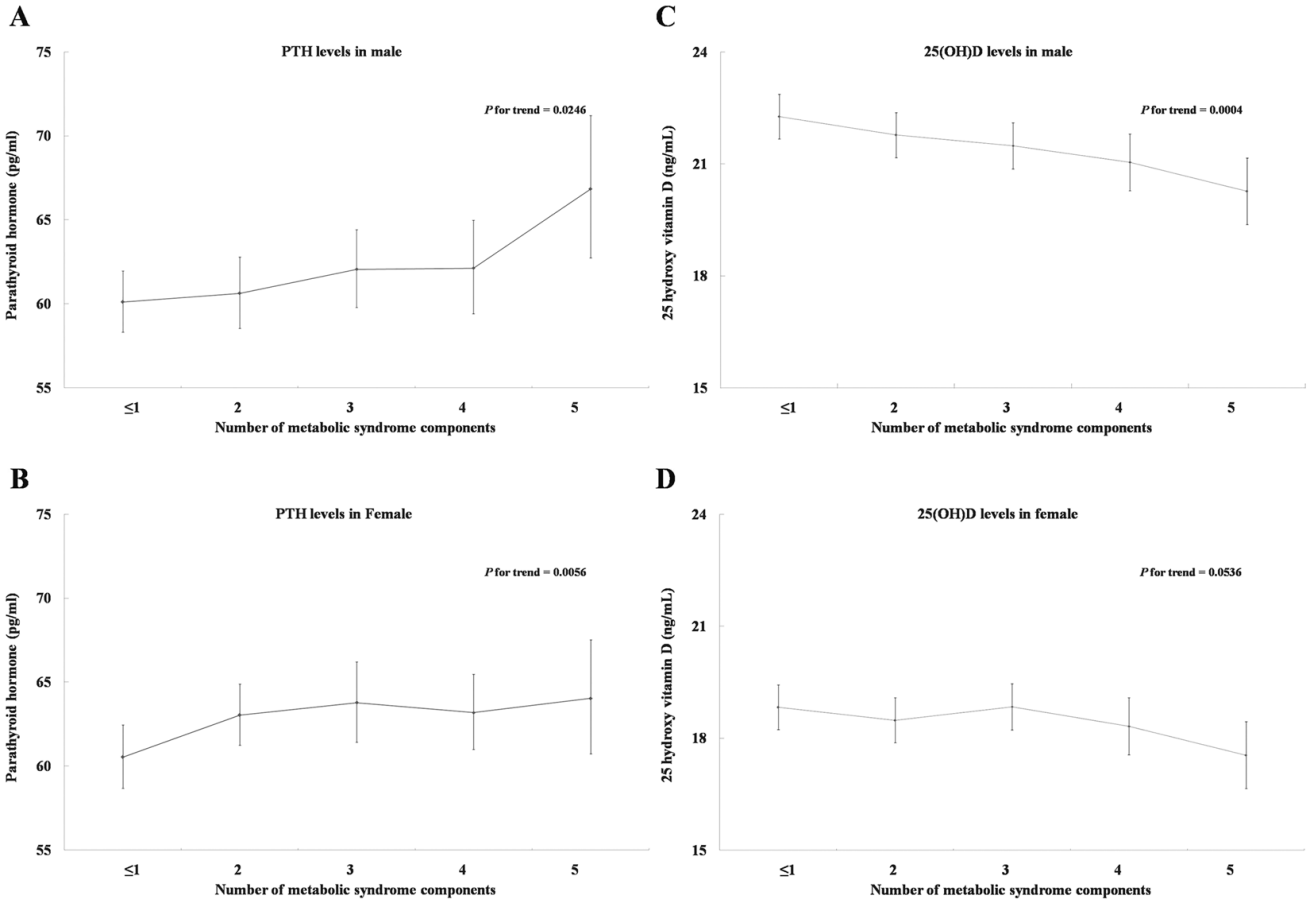
PTH (**A** and **B**) and 25(OH)D levels (**C** and **D**) according to the number of metabolic syndrome components.

While significant decreases in 25(OH)D levels were observed only in male subjects, an increase in PTH levels was observed for both genders with an increase in the number of MetS components (Fig. 1).

## Discussion

A significant association of PTH and vitamin D with MetS was found in an analysis of representative nationwide data of the middle-aged Korean population with preserved renal function. Specifically, PTH levels in females and vitamin D levels in males with MetS were significantly different from those of subjects without MetS after adjustment for possible confounding factors. The association patterns of PTH and vitamin D with each component of MetS were different between males and females. In addition, significant increases in PTH levels in both genders and a decrease in vitamin D levels in males were observed based on the number of MetS components present.

In terms of the association of PTH levels with MetS, our study showed similar results to previous studies^6,8^, which excluded subjects with impaired renal function. Even in a study of obese populations, which did not exclude CKD subjects^10^, an increase in PTH was associated with the presence of MetS after adjustment for confounding factors including the GFR. However, although an association of PTH levels with MetS was found in our study, statistically significant differences after adjusting several confounding factors remained only for females, unlike prior studies that showed a significant relevance in men^6,8^ or in both genders^10^. Some studies failed to show a relationship between PTH and MetS in large population datasets^9,14,15^. Among them, a study by Ford *et al*.^9^ included relatively young subjects with a mean age of under 50 years and no adjustment of GFR or exclusion criteria for CKD subjects. More than 50% of the subjects were under 50 years old, and the incidence of MetS was less than 30% in a study by Li *et al*.^14^. As mentioned previously, a recent study using data from the KNHANES^15^ that found higher levels of PTH than our study did not consider the effects of the GFR on PTH and vitamin D in the analysis. An association of vitamin D with MetS was observed for males in our study. Among previous large-scale population studies, some have shown an inverse relationship between vitamin D status and the presence of MetS^7,8,11,13,15,26,27^, but others did not show this association^6,28,29^. Four studies^7,11,13,26^ showed significant associations of vitamin D with MetS but did not measure the levels of PTH. Inconsistent results regarding the association of PTH and vitamin D levels with MetS may be caused by several factors, including whether the GFR was considered in the analysis. As differences in vitamin D metabolism have been observed among various ethnicities^30^, and a prior study examined the effect of ethnicity on the risk of MetS^29^, the effect of the ethnicity of the studied population should be considered.

The mechanism by which PTH and vitamin D affect the development MetS has been explained in several ways. As summarized in a review article^31^, PTH could increase intracellular calcium in skeletal muscle and adipocytes, which would consequently suppress glucose uptake by those tissues and induce insulin resistance. In a recent study, increased lipolysis induced by PTH could cause insulin reisstance^32^. Additionally, a prosclerotic effect on vascular smooth muscle cells^33^ has been suggested. Likewise, vitamin D deficiency also harbours a possible mechanistic link to the development of MetS because it influences insulin secretion^34,35^, adipogenesis^36^ and renin-angiotensin system^37^.

The gender difference in the association of PTH and vitamin D with MetS is not fully understood. Differences in the prevalence of vitamin D deficiency in males and females^38^ may contribute to the different degrees of association of PTH and vitamin D with MetS. Importantly, vitamin D deficiency is prevalent in the Korean female population^39^. As oestrogen could act as a stimulator for calcium absorption in the intestinal tract^40,41^, oestrogen deficiency in females may contribute to the difference in association patterns that we observed. Although no detailed data for hormone replacement in postmenopausal women was obtained, most of the female subjects would likely have oestrogen deficiency considering the mean age of the studied female population (61.7 ± 0.2 years).

Based on the results, gender specific application of both hormones might be considered as a biomarker or therapeutic target of MetS. PTH in females could be suggested as an additional surrogate marker for development of MetS. Though it would not be possible to provide a certain cut-off values of PTH in terms of increasing MetS, at least, increasing PTH with normal calcium and vitamin D homeostasis might be warning sign for MetS in females. In addition, it would be very interesting to elucidate the role of intervention, that is supplementation of calcium and vitamin D for female with higher PTH and for male with lower vitamin D, to prevent MetS. There is no interventional study to identify the role of calcium supplement in prevention of MetS yet. Though possible benefit of vitamin D supplement on glycemic control or insulin resistance was suggested^42^, it is not conclusive^43^. Therefore, further well-organized studies are required to determine the MetS preventive effect of calcium and vitamin D.

Interestingly, according to our results, as the number of MetS components increased, the levels of PTH increased in both females and males (*p* for trend 0.0056 in females, 0.0246 in males). A statistically significant decrease in vitamin D levels was observed only in males (*p* for trend 0.0004). From a clinical perspective, elevated PTH levels may be a useful surrogate marker for the risk of MetS in subjects with preserved renal function.

Several limitations could be noted in our study. First, no causal relationship could be deduced due to the cross-sectional design of the study. Second, no detailed data were provided on the supplementation of calcium and vitamin D (i.e., dose) in the studied subjects, and the effect of supplementation could not be estimated. Third, subjects with primary aldosteronism or renovacaulr hypertension, which would lead to secondary hyperparathyroidism and increase the risk of Mets^44^, could not be identified and excluded. Lastly, information about sun exposure and seasonal variation, which are related to the status of vitamin D, was unavailable for the analysis.

In this study, we excluded subjects with impaired renal function for several reasons. First, PTH and vitamin D levels are closely related to the GFR^45^. Specifically, different patterns (steep increases) of PTH changes according to the GFR were noted in subjects with stage 3 CKD, and subjects with stage 3b CKD demonstrated an approximate 5-fold increased risk of high PTH (70 pg/ml) compared to individuals with a GFR ≥ 60^16^. In contrast to stage 3a CKD, stage 3b patients exhibit a higher rate of progression to end-stage renal disease^46^ and a  higher mortality rate^47^. Moreover, stage 3b CKD patients have been shown to exhibit more metabolic complications than stage 3a patients^48^. In addition to the relationship between PTH and GFR, vitamin D deficiency, which is also related to PTH secretion, is prevalent in subjects with CKD^49,50^. Second, considering the current management guidelines of CKD patients^17^, the likelihood of patients undergoing vitamin D and calcium supplementation, which would interfere with the levels of PTH and vitamin D, would be high. Lastly, CKD itself is also a well-known risk factor for MetS^18,51–57^. Therefore, it would be reasonable to evaluate the association of PTH and vitamin D with MetS in subjects with preserved renal function separately from those with moderate to severe CKD. In a recent KNHANES study^15^ that evaluated the association of PTH and vitamin D with MetS, different results from ours were obtained. This discrepancy may have occurred because the GFR was not considered in the analysis of the former study. Because no exclusion criteria for CKD were applied, the prior study^15^ presented higher mean levels of PTH (approximately 70 pg/mL) than our study.

In summary, in a study of nationwide representative data of a middle-aged population with preserved renal function, gender-specific associations of PTH and vitamin D with MetS were found. PTH exhibited a possible link to MetS in females, whereas vitamin D showed a possible link to MetS in males. The number of MetS components was related to an increase in PTH and a decrease in vitamin D levels. Based on the results of this study, further investigations that are especially focused on gender specificity should be conducted to identify the mechanism that links these hormones to MetS.
